# Collectanea

**Published:** 1828-07

**Authors:** 


### COLLECTANEA.

Floriferis ut apes in saltibus omnia libant,

Omnia nos, itidem, depascimur aurea dicta.

### ANATOMY.

*Termination of the Stomach in a Cul-de-Sac.*—A CHILD, born at the eighth month of pregnancy, presented externally a natural appearance. Six hours after birth, it vomited a considerable quantity of dark fluid, resembling half-coagulated blood. It died in about sixty hours. The stomach was found much distended, and occupying nearly the whole of the left side of the abdominal cavity. Its internal surface was spongy and inflamed, and black in different parts. There were also several bubbles of air, which were probably produced by gangrene. The pyloric extremity communicated with a pouch, or second stomach, in which the only opening that existed was the duct of the pancreas. There was no communication whatever between the stomach and intestines. The intestines were shrunk, and lay between the stomach and liver, in an agglomerated mass.—*Med. Zeitung.*

### PHYSIOLOGY.

*On the Influence of the Pneumo-Gastric Nerves.*—Mr. E. WARE observes, that there is nothing more frequently praised than the certainty of the evidence of natural truths. Each writer in his turn thinks his discoveries completely established, and arrays his alleged facts in an apparently resistless phalanx. But almost every one who elicits novelties is, in spite of his confidence, subjected to controversy and contradiction. His facts are denied, his experiments refuted, his conclusions declared inadmissible, and his veracity and philosophical honesty called into question. These reflections were suggested by a perusal of the written conflict respecting the influence of the eighth pair of nerves on digestion. The experiments of WILSON PHILIP illustrative of the influence of the eighth pair of nerves on respiration and digestion, have been subsequently repeated by an English writer, BROUGHTON, and results diametrically opposite been reported. In order to satisfy myself as to the cause of such discrepancy, and, if possible, to ascertain the truth, free from all bias or prepossession, I instituted the following course of experiments, assisted by Dr. J. H. FINLEY, and others of my medical friends.

Experiment 1.—After causing two rabbits, of the same age and size, to fast for the space of sixteen hours, I gave them as much parsley as they would eat. One of them I set at liberty. In the case of the other, I divided the nerves of the eighth pair, about midway in the neck. The division was immediately followed by difficult respiration, soon attended with a croaking noise, and gradually increased until death supervened; which happened in six hours and a half after the operation. The other rabbit was then killed, and a comparative examination made. The stomach of the one subjected to the operation was much distended; the general mass of food had undergone but little change: that part which was in contact with the parietes of the stomach was altered in colour, and somewhat in consistence, resembling partially digested matter. The central parts retained their natural colour and odour, and resembled finely chopped parsley. The lungs were largely engorged with blood, but did not sink in water. The trachea and air cells contained a frothy fluid.—The stomach of the rabbit not operated on was hard and contracted, and about half the size of that of the other. That part of digestion confided to the stomach was comparatively completed; for though all the contents had not passed out at the pylorus, yet what remained was a uniform chymous mass, more compact and dry at the pyloric than towards the central or cardiac portions.—This experiment was satisfactory and conclusive, and was followed by six others of a similar nature, all of which gave uniform results.

Experiment 2.—A half-grown cat was kept without food for twelve hours, and then one ounce and a half of raw beef was given. In fifteen minutes after, I divided the eighth pair of nerves. The usual symptoms followed, differing from those of the last experiments only in degree: respiration was deep, slow, and laborious, attended with a croaking noise, and apparent efforts to vomit, which continued to increase for ten hours, when, from the great distress and prostration, I was induced to kill it. Upon examination, no change was perceptible in the food, except in the circumference, which had lost its livid hue, and resembled beef shortly after being placed in warm water. It had lost nothing in weight. The lungs were engorged with blood, and the trachea filled with frothy fluid slightly tinged with blood.—This experiment was also confirmed by one other.

Experiment 3.—Having caused two rabbits, of the same age and size, to fast for sixteen hours, I allowed them as much cabbage as they could eat, after which I made a section of the eighth pair of nerves in each. One I set at liberty. The other, the hair being shaved, on either side, from the region of the stomach, I submitted to the influence of a galvanic trough, containing fifty pair of four-inch plates; the intervals being filled with sulphuric acid and water, in the proportion of one of acid to sixty of water. A gentle and uniform twitching of the muscles of the trunk was kept up, by the occasional addition of acid, for six hours; at the end of which time (the animal being almost exhausted) it was killed by a blow upon the occiput. Examination being made, the stomach was found distended as in the preceding experiments; the external part of the contents was changed in colour and somewhat in consistency, so as to resemble a chymous mass; whilst internally it was as if it had been chewed and swallowed. The lungs were engorged, though not quite so much as usual. During the process, the respiration exhibited the phenomena seen in the other cases. The trachea contained a frothy fluid. This animal ate nothing after the operation, and nothing was found in the œsophagus.—In half an hour after, (six hours and a half after the operation,) the other rabbit died: the stomach and its contents resembled in every respect that of the galvanised rabbit. The lungs were more largely engorged with blood, which constituted the only apparent difference.

Experiment 4.—A rabbit, after fasting, was afforded as much parsley as it would eat; when the hair was shaved on the back, near the region of the stomach, and a small plate of tin bound thereon: the eighth pair of nerves were divided, and about a quarter of an inch of the lower section of each coated with tin foil. The tin foil and tin were connected with the opposite poles of the galvanic trough, and a uniform effect kept up for five hours and a half, when the animal died. Its respiration during the process, the state of its stomach, food, lungs, and trachea, differed in no particular from those in the immediately preceding experiments.

Experiment 5.—Two rabbits were caused to fast for twelve hours, when there was given as much cabbage as they would eat. The one remained in its natural state. In the other, the pneumo-gastric nerves were divided, and submitted to the galvanic power as in the last experiment, and a uniform effect was kept up for seven hours, when the animal died. Upon examination, it was found to differ in no perceptible degree from those in which the nerves had been divided. Whilst the stomach of the healthy rabbit exhibited the contents diminished in quantity, and in a completely chymous state, and in the pyloric portion comparatively dry and compact.

These experiments, made with much care, time, and labour, serve to show the great caution with which we should receive the accounts of even experimental inquiries. They afford results agreeing with those of Dr. Philip, respecting the effect on digestion by the division of the par vagum; for by that section digestion is almost totally arrested; and, although they would indicate partial action, it is so slight that it may be justly attributed to the healthy secretions of the stomach poured out immediately preceding the operation. Although secretion, the alleged cause and, as we believe, the *primum mobile* of chymification, does not cease after the division of these nerves, but, on the contrary, appears to be augmented, yet, inasmuch as it is incapable of changing food to chyme, it must be morbid. Another apparent barrier to chymification is the deficiency of action in the muscular coat of the stomach, known from the violent and ineffectual efforts to vomit,—symptoms constant and remarkable, particularly in cats,—and as proved in those rabbits which were permitted to eat after the operation, where food was found in the œsophagus itself. But when we endeavour to institute the galvanic irritation for that action carried on through continuous nerves, we cannot confirm either the facts or conclusions of Philip. Our results are entirely contradictory of his: and we are compelled to acknowledge our belief in the inaccuracy of his galvanic experiments, and stand prepared to deny that there is yet any good reason for believing in the identity of galvanism and the mysterious principle of life.

Death, in all the cases here related, seemed to be caused rather by the state of the lungs than by that of the stomach; for the engorgement and obstruction in the organ of respiration put finally an entire stop to the access of air into the bronchial cells, and suffocation ensued.—*North American Medical and Surgical Journal.*

---

### PATHOLOGY.

*Chronic Inflammation of the Arteries.*—A lady, sixty-five years of age, who had been subject to frequent headache, and rheumatic and gouty pains, was attacked, during the latter period of her life, with the following symptoms: From the commencement until the time of her decease, her pulse was very irregular. Frequent attacks of dyspnœa, which were only relieved by bleeding. Urine scanty, of a red colour, and turbid. Pain in the præcordia. Numbness of the whole of the left side. Violent pains in the limbs, which were alleviated by leeches. After death, the internal membrane of the aorta was found in a highly inflamed state, especially at the points where any small arteries branched off. There were also various spots of ossification in the course of the artery.—*Med. Chir. Zeitung.*

*Observations on some Points of Pathology.*—Dr. Horner states, that a fine injecting matter may be pushed into any vessels into which red particles of blood can naturally penetrate. He has repeatedly filled the whole venous system from the arterial, so as to display all the fine venous meshes under the skin, and to infiltrate the body completely. Judging from these experiments, he is disposed to think that some of the phenomena of inflammation arise mechanically, and that the substance effused from vessels is in a measure according to the mass and momentum of blood flowing through them. Thus, when irritation determines an increased afflux of blood to a part, if the calibres of vessels are not large enough to permit it to pass freely from the arteries into the veins, serous infiltration first of all occurs: if the afflux be augmented, then coagulating lymph, the particles of which are larger, is effused; and if there be a further augmentation of afflux, the red particles of blood are then effused through the lateral porosities of the vessels. The corresponding phenomena in fine injections are, first the water, then the size, and lastly the colouring matter, from its particles being the coarsest of the mixture.

Though many dropsical effusions may be traced to irritation, yet he is disposed to think that some very great errors have been incorporated with their pathology, from the desire to adapt all the phenomena to one standard,—to wit, inflammation. This at least he is convinced of, that in fine injections of whole adult dropsical subjects, no resistance scarcely is offered by the blood-vessels, and that the injected fluid escapes from them by their lateral parietes or porosities, as fast as it can be thrown in; manifesting thereby evidently a great laxity in their texture. This escape is generally in the order in which we see dropsies occur,—first in the ankles and feet, then up the lower extremities to the trunk; in the hands and wrist, and then up the pectoral extremities to the thorax.

The purpura urticans which occurs in the skin in dropsy, seems to be an extravasation of blood arising from the same passive or loose texture of the blood-vessels. He has repeatedly seen the same sort of ecchymosis in the muscles of dropsical subjects, and in the interstices between them.

Pathologists have said much on the distinction between venous and arterial capillary hemorrhage: he doubts very much the practicability of making out either case very clearly and distinctly from the other, yet they have rather unsoundly drawn the inference, that spontaneous bleeding from the venous system is passive, while that from the arteries is active. Judging from mechanical arrangement, he is inclined to think that all hemorrhage, not arising from violence or mechanical injury, comes from the arteries; inasmuch as microscopical observation teaches us that the blood, in getting into the extreme arteries, is there confined to the smallest sanguiferous channels, and it is of course there that rupture is most likely to occur; for, so soon as the blood reaches the veins, the channels are larger, more dilatable, and therefore less liable to be broken. Genuine venous hemorrhage is probably most frequently the result of obstruction to a large venous trunk, either by pressure on it or by something similar: thus we see in women, during pregnancy, bloody spots of ecchymosis on the legs from ruptured veins; and, in persons with varicose ulcers, frequent bleeding from the latter, when the erect position puts too great a column of blood upon them.

Red blood is excreted from the pleura in its severe inflammations; and, judging from what he has observed, the order of the effusion is first serum, then coagulating lymph, and then red particles of blood, all of which may, as in inflammation of other textures, be indicative of the gradual dilatation of the blood-vessels. The most remarkable instance of this successive secretion, excretion, or effusion, whatever it may be called, that he has met with, occurred the last winter in the Pennsylvania dissecting rooms: in this case the marks of inflammation were all recent, and had supervened upon a tuberculous phthisis of both lungs. When inflammation occurs in the pulmonary tissue itself, it is always marked by an accumulation of red blood in their structure, giving them a red purple colour, (BICHAT, Anat. Gen. vol. ii. p. 62,) by their increased weight, diminished elasticity, and by their being much more watery than usual, probably from the serum being separated from the red blood after death. There is also a considerable quantity of frothy mucus in the bronchia. This state of the lung is most frequently attended with a recent effusion of coagulating lymph on the pleura, and by a bloody serum in the thorax. It is, however, to be observed, that bloody serum being found in the cavity of the pleura is not an absolute proof of its being secreted or discharged in that state, because if the examination be much postponed after death, the serum effused may be tinged by the lung soaking in it.

It is owing to the blood-vessels of the lungs being so superficial, that, as in the intestines, their inflammations pass off either by an increase of their natural secretion of mucus, or by the effusion of serum and of blood. LAENNEC has said (vol. i. p. 116,) that a collection of pus in the pulmonary tissue, in consequence of inflammation, is one of the rarest of cases,—at least it is one hundred times more rare than a vomica from tuberculous matter, and a thousand times more so than empyema. In all the dissections of lungs that he has made, he has met with it but once, and that lately, (June 29th, 1827,) at the Almshouse, in which case the surrounding part of the lung was gangrenous.—*American Journal of Medical Sciences.*

---

### PRACTICAL MEDICINE.

*Chronic Bronchitis treated by the external Application of the Acetate of Morphia.*—A woman, fifty-five years of age, of a nervous and delicate constitution, had suffered a long time from a catarrhal affection, after having had several attacks of bronchitis. The symptoms were particularly severe at the menstrual periods. Fourteen years had elapsed since the commencement of the malady, and no benefit had been derived from the various modes of treatment that had been tried. She was much emaciated, had constant cough, with thick greyish expectoration. A sensation of itching along the trachea much annoyed her. By percussion and auscultation, it was ascertained that the lungs were not organically diseased. From the failure of all the previous efforts to relieve her, a new mode of treatment was determined upon. The bowels having been constipated for some days, five grains of powdered aloes were placed upon a blister, which had been applied to the left arm. Ten hours afterwards, she had several motions, and slight colic. Half a grain of acetate of morphia in fine powder was next applied upon the blistered surface. In a quarter of an hour, the itching of the trachea, the cough, and oppression of the chest, were diminished. On the following day, one grain of the same medicine was applied in a similar manner. In the course of an hour, the patient felt herself decidedly relieved, and she enjoyed some sleep, which she had not done for a long time before.

The dose of the morphia was gradually increased to two grains, and in a short period the cough was nearly cured. To ascertain positively whether the relief had depended upon the effects of the medicine, it was discontinued, and all the symptoms quickly returned. To prevent the possibility of the mind of the patient having any influence, in consequence of which a false opinion would have been formed of the effect of the morphine, she was frequently deceived. It was very evident, however, that the amelioration of the symptoms was owing entirely to the use of the morphine.

By slow degrees, the quantity was increased to four grains, and from this period she daily recovered. She gained flesh, and presented a healthy appearance.—LEMBERT, *Méthode Endermique.*

---

*Use of Gymnastic Exercises in Chorea.*—M. LOUVET LAMARRE has lately published a case tending to prove that gymnastic exercises, judiciously employed, are of use in this disease. A young lady had had two or three paroxysms each year for several years. Each paroxysm lasted "*plusieurs mois.*" The convulsions were so violent that she was obliged to be assisted in eating. The superior extremities were the parts principally affected, and these were thrown into the most sudden and irregular motions. Her sleep was much disturbed. Leeches were applied behind the ears, the warm bath was used, and the spine was rubbed with camphorated liniment. At the same time the patient was desired to exercise herself with a skipping-rope, as long as she could bear the exertion. An amendment soon took place. Her general health improved. At first, the convulsive motions ceased only after she had exerted herself with the rope, but they soon disappeared entirely. The attack lasted but twenty days.—*Nouvelle Bibl. Med.*

In all convulsive affections, in which the patient retains his mental faculties, it is important to abstract the attention as much as possible from the state of the affected parts. Hiccup is often relieved by mental excitement. We have at this moment a young lady under our care, who suffers severely from chorea; but, if her mind is actively employed in any amusement, the symptoms disappear.

Professor CRUVEILHIER adopted an ingenious expedient in a case of traumatic tetanus, which has some analogy with the suggestion of M. Louvet Lamarre. The patient was afflicted with the most severe and alarming convulsive action of the diaphragm. He was induced to take very deep and measured inspirations. The convulsions abated, and soon ceased entirely.

At the Salpêtrière, chorea is considered so trifling, that no remedy is employed. As the disease, however, frequently depends upon some obvious derangement of health, such negligence is not to be justified.—E.

---

*Practical Observations on the Datura Stramonium.*—Dr. CUNNINGHAM gives the following as the results of his experience with regard to stramonium.

The first disease in which I used the plant was the *asthma.* During the paroxysm of this distressing complaint, I have directed the seeds to be smoked until vertigo was produced, which almost invariably terminated the paroxysms. The beneficial effects of the plant, thus used, induced me to try its efficacy in preventing the attack. I accordingly directed my patients to smoke the seeds until vertigo was produced, whenever they felt the premonitory symptoms of the disease: this has uniformly prevented its occurrence. In several cases I have reason to believe that a complete cure has been effected. In the summer of 1818, I was called to see a woman, labouring under a violent attack of this disease. The smoking of one seed removed the existing paroxysm. She then informed me, that for fifteen years she had been afflicted with the disease; that it was brought on, as she believed, by a sudden cessation of the menses, from exposure to severe cold. For several years the attack was monthly; it then came on every two weeks; and, for the last two years, she has had an attack every week. Generally the paroxysm is slight, especially in the summer season, but occasionally it is very violent, and frequently continues, especially in the winter season, for two or three days. In this case, with but a faint hope of a final cure, I directed the remedy to be used whenever she felt any symptoms of an attack. Six months after this time I again saw this patient. She had succeeded, by means of the remedy, in preventing any recurrence of the disease. As above stated, one seed produced vertigo when first used; now she smokes a pipe full. In the fall of 1819, I once more saw this woman: she still continued free from the disease, and had not for several months used the medicine.

In *epilepsy,* although I cannot relate any cure, still I have realised the happiest effects from the stramonium. Twenty grains of the powdered leaves, three times a day, and of the saturated tincture, made of the bruised seed, a teaspoonful every six hours, were taken by a patient of mine, a boy fourteen years of age. Previous to his taking the medicine, he had from ten to thirty fits every twenty-four hours. The medicine produced first an abatement of the violence of the fits, then of their number, and, when the ultimum dose was attained, a complete cessation of the fits for six weeks. The medicine was omitted by the friends of the boy: the fits returned, and proved fatal. Notwithstanding the disease in this instance had existed for eight years, and had attained to a great degree of violence, still, from the effects produced by the medicine, I was induced to hope for a favorable termination, if the remedy had been persevered in. The medicine in this case operated freely on the bowels, and the morning doses produced dilatation of the pupil, which continued but for a short time.

The stramonium will be found a valuable remedy in *inflamed ulcers,* and in those ulcers attended with great irritation, for which the Conium Maculatum has been found so highly useful. The bruised leaves may be applied, or, what in some cases answers a better purpose, the leaves wilted by pouring boiling water over them, and applied warm, will prove a very soothing application.

In *inflammation of the mammæ,* I have found the warm stramonium poultice a valuable discutient.

In *hemorrhoids,* the bruised leaves in some cases, in other cases the warm poultice, have proved the most valuable remedy I have ever tried.

The disease to which I wish most particularly to call the attention of my medical brethren, and for which the stramonium is the most valuable remedy that I have known to be used, is a sequela of the fevers of southern climates and an almost universal attendant of the intermittents of this section of country. I mean the *enlargement of the spleen,* or ague cake, as it is generally termed. The immense size which this viscus attains is hardly known to practitioners in northern climates. The rapidity of its growth is truly astonishing; and although it occasionally, without any direct means, diminishes in size, so as to be no longer troublesome to the patient, it generally proves very difficult to remove, and is very grievous to the person afflicted therewith. I first saw this disease in Batavia, in the year 1804; the treatment I then recommended was that usually prescribed for chronic hepatitis. Since my residence in this part of our country, I have had frequent opportunities of treating this disease in the manner thus practised. In some cases I have been successful, in many I have failed. For the last three years I have had recourse to the stramonium. Externally applied, I am warranted in saying that the leaves, wilted by boiling water, and applied as a poultice over the enlarged spleen, as warm as the patient can bear, and renewed night and morning, will, if persevered in, completely remove the disease. It will be necessary to keep the bowels open, by taking a small portion of Epsom salts every night. By this means I have not failed to remove the disease in a few days, in recent cases: those of long standing will require perseverance in the remedy for several weeks. In the following cases, a small variation in the treatment was rendered necessary from peculiar circumstances.

On the 9th of September last, I was called to see G. D., a man of thirty-five years of age, and found him labouring under an enlargement of the spleen, which extended nearly down to the ileum, and across the abdomen, two inches to the right of the umbilicus, and (as well as I could judge) the thickness was fully three inches. The disease had existed for several years, but had given no particular uneasiness except from its bulk, until four days ago, when inflammation, accompanied with great pain and soreness of the abdomen, had ensued. He had bled himself freely, and had applied a large blister, without relief. The pain was very acute at night; in the morning it abated.

> ℞. Sal. Epsom. ℥iij.; Sal. Nitri. ʒij. Mix; dissolve in ℥viij. of water. A tablespoonful to be taken every hour, until the bowels are freely operated upon.—Afterwards take a pill containing three grains of opium and two grains of tartar emetic. These medicines to be continued every day, until the pain be removed.—The stramonium poultice to be applied night and morning.

On the 16th, I again saw my patient. The spleen was no longer to be felt, the abdomen free from pain and soreness. Colic pains were troublesome for several days after this time.

On the same 9th of September, I was called to a child of R. W., three years old. She complained of pain and soreness of the belly; the spleen occupied as much space as in the former case. There being some symptoms of worms, I directed the following combination:

> ℞. Calomel gr. vj.; Gambogi gr. vj. Mix; divide into six equal portions; one to be taken night and morning in syrup.—The stramonium poultice to be applied night and morning.

On the 16th, the child was free from complaint; the spleen not to be felt. —*North American Med. and Surg. Journal.*

